# A Patient Affected with Serous Ovarian/Peritoneal Carcinoma Carrying the *FANCM* Mutation

**DOI:** 10.1155/2019/9357924

**Published:** 2019-05-16

**Authors:** Adamantia Nikolaidi, Irene Konstantopoulou, Nikolaos Pistalmantzian, Florentia Fostira, Drakoulis Yannoukakos, Ilias Athanasiadis

**Affiliations:** ^1^Oncology Department MITERA Hospital, Athens, Greece; ^2^Molecular Diagnostics Lab, NCSR “Demokritos”, Athens, Greece

## Abstract

We report a case of a 58-year-old female with ovarian cancer. The patient presented with ascites, and the biopsies revealed a low-grade adenocarcinoma, either a serous papillary ovarian cancer with peritoneal implants or a primary peritoneal carcinoma. She received neoadjuvant chemotherapy and after 5 cycles achieved partial response, and then, she underwent a total hysterectomy/bilateral salpingo-oophorectomy. The patient underwent germline gene-panel testing for the detection of mutations in cancer predisposing genes. A truncating mutation in the Fanconi anemia complementation group M (FANCM) gene was detected in heterozygosity, namely, p.Arg658Ter (c.1972C>T, rs368728266). The patient's family history is unremarkable, with no reported cases of breast or ovarian cancer, a fact that can be attributed to the significant lower penetrance of FANCM mutations.

## 1. Introduction

A 58-year-old Greek woman presented with abdominal distension and discomfort. A CT scan was performed on 12/04/2017 depicting ascites, enlarged pelvic lymph nodes, and mesenteric fat turbidity. An abdominal MRI confirmed those findings and revealed intraperitoneal implants of the mesentery. Tumor marker Ca 125 was measured at 339.10 U/ml.

Biopsies were performed on 26/04/2017, and histology examination revealed a low-grade adenocarcinoma. Immunohistochemistry was positive for WT1, ER, and MOC31 and negative for p53, calretinin, and D240, suggesting either a serous papillary ovarian carcinoma with peritoneal infiltration or a primary peritoneal carcinoma.

The patient was administered cytotoxic chemotherapy with the combination of paclitaxel (175 mg/m^2^) and carboplatin (AUC 6), resulting in partial clinical and biochemical responses with decreasing ascites formation and tumor marker Ca 125, which measured at 91.6 U/ml within a two-month period.

Following that, the patient underwent a total hysterectomy/bilateral salpingo-oophorectomy on 27/9/2017 and, according to the pathology report, an R1 resection.

One week after the surgery, the patient presented acute peritonitis due to anastomotic rupture and was surgically treated with an ileostomy formation. The patient remained hospitalized for two months because of postoperative complications (electrolyte imbalance, infected intra-abdominal hematoma).

The last imaging test was performed on 12/1/2018 and depicted stable disease, while at the same time, Ca 125 values were within a normal range.

Following the national guidelines for ovarian cancer, the patient underwent germline gene-panel testing for the detection of germline mutations in 94 cancer predisposing genes (Illumina TruSight Cancer panel). While no *BRCA1/2* mutations were found, a truncating mutation in the Fanconi anemia complementation group M (*FANCM*) gene was detected in heterozygosity, namely, p.Arg658Ter (c.1972C>T, rs368728266).

*FANCM* is one of the nearly 20 genes implicated in the major DNA repair pathway of interstrand crosslinks through homologous recombination (HR). The major breast and/or ovarian cancer predisposing genes *BRCA1* and *BRCA2* are also members of this pathway, while biallelic mutations in genes of the HR pathway cause Fanconi anemia (FA), a genetically heterogeneous recessive disorder characterized by cytogenetic instability, hypersensitivity to DNA crosslinking agents, increased chromosomal breakage, and defective DNA repair [1].

Monoallelic mutations in FANCM, as well as in other FA/HR genes, have been recently linked to breast cancer predisposition, conferring an approximately 2-fold increase in lifetime risk for the disease and showing a stronger correlation with the triple-negative phenotype (OR: 3.75) [2]. These findings identify FANCM as a breast cancer susceptibility gene [3]. Although still preliminary, a role for *FANCM* as an ovarian cancer susceptibility gene has very recently emerged [4]. It has to be noted however that dysfunction of the HR mechanism has been strongly linked with breast and ovarian cancer predisposition, even though the exact role of each protein involved remains to be scrutinized with respect to magnitude risks and possible therapeutic implications.

**Figure 1:**
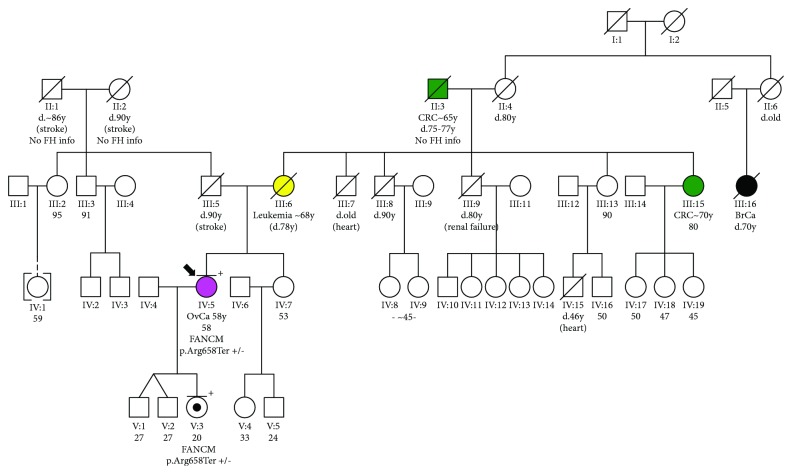
Pedigree of the patient carrying the loss-of-function monoallelic FANCM mutation p.Arg658Ter. OvCa: ovarian cancer; CRC: colorectal cancer; BrCa: breast cancer.

The patient's family history (Figure 1) is unremarkable, with no reported cases of breast or ovarian cancers. This however can be attributed to the lower penetrance of *FANCM* mutations, compared to that of the major breast/ovarian cancer predisposition genes. The scarcity of females on the paternal side should also be taken into account. The 20-year-old daughter of the patient also carries the p.Arg658Ter mutation. This case is the first case of ovarian cancer with FANCM mutation in Greece.
